# Metachronous metastatic paraganglioma in jejunum as a rare entity: A case report

**DOI:** 10.3892/ol.2015.2860

**Published:** 2015-01-09

**Authors:** HUSEYIN KAZIM BEKTASOGLU, GOKHAN CIPE, ERKAN YARDIMCI, DILEK SEMA ARICI, MUSTAFA HASBAHCECI, OGUZHAN KARATEPE, MAHMUT MUSLUMANOGLU

**Affiliations:** 1 Department of General Surgery, Faculty of Medicine, Bezmialem Vakif University, Istanbul 34093, Turkey; 2 Department of Pathology, Faculty of Medicine, Bezmialem Vakif University, Istanbul 34093, Turkey

**Keywords:** paraganglioma, pheochromocytoma, jejunum, bowel obstruction

## Abstract

Pheochromocytomas and paragangliomas are neuroendocrine tumors that arise from chromaffin cells of adrenal medulla and extra-adrenal paraganglia, respectively. The recurrence of these neuroendocrine tumors as a jejunal mass causing obstruction in the small intestine is an exceptional entity. The present study reports the case of a 70-year-old male who presented to the Emergency Department of Bezmialem Vakif University Hospital with abdominal pain and vomiting. The patient possessed a history of left nephrectomy due to malignant pheochromocytoma that had invaded into the left kidney eight months prior to presentation. Bowel obstruction was diagnosed and the patient underwent a laparoscopic procedure. Partial resection of the jejunum was performed and immunohistochemical studies revealed the lesion to be malignant paraganglioma. The majority of paragangliomas are chemo- and radioresistant. Surgical excision remains the primary treatment. Metachronous paraganglioma arising from the small intestine is an extremely rare entity and may be a relevant consideration in patients presenting with bowel obstruction.

## Introduction

Pheochromocytomas and paragangliomas are neuroendocrine tumors that arise from the chromaffin cells of the adrenal medulla and extra-adrenal paraganglia, respectively. Pheochromocytomas are occasionally termed intra-adrenal paragangliomas (1). The annual incidence rate of paraganglioma is ~1.5 cases per million individuals worldwide, and the peak incidence occurs between the ages of 30 and 50 years (2). Paragangliomas may be derived from the parasympathetic or sympathetic ganglia. The majority of paragangliomas derived from the parasympathetic ganglia occur in the head and neck, while the majority of paragangliomas derived from the sympathetic ganglia occur in the abdomen, adjacent to the adrenal glands (1). However, paragangliomas may occur in any region where the sympathetic or parasympathetic ganglia exist. Despite the majority of paragangliomas being sporadic, a genetic association has been identified in <50% of cases due to the evolution in genetic testing (3). In total, ~25% of paragangliomas are malignant and metastasis may arise ≥20 years following the first presentation (4). Due to the difficulty of confirming malignancy, all paragangliomas should be considered malignant. The only definitive sign of malignancy is distant metastasis to organs such as the bone, liver and lymph nodes (2). Due to the majority of paragangliomas are chemo- and radioresistant, surgical excision of the tumor is the primary treatment (2,5,6). The five-year survival rate of patients with malignant paragangliomas is ~60% (6). The present study reports a case of a jejunal mass resulting in an obstruction that was treated laparoscopically and diagnosed as malignant paraganglioma. The patient possessed a history of left nephrectomy due to malignant pheochromocytoma that had invaded into the left kidney eight months prior to presentation. Written informed consent was obtained from the patient.

## Case report

**Figure 1:**
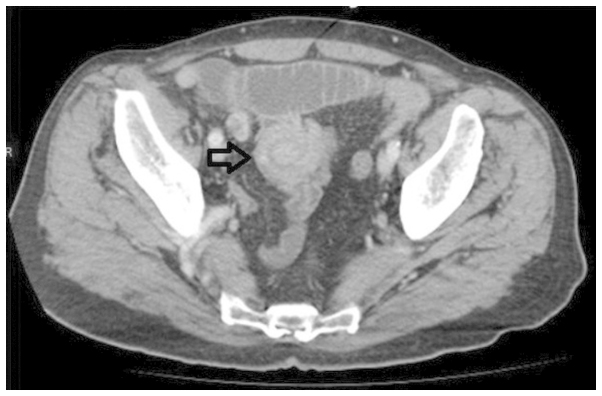
Computed tomography revealing a contrast enhanced mass (black arrow), which resembled a neuroendocrine tumor.

The present study reports the case of a 70-year-old male that presented to the Emergency Department of Bezmialem Vakif University Hospital (Istanbul, Turkey) complaining of abdominal pain, a lack of defecation for one week and vomiting of two-day duration. The medical history of the patient included left nephrectomy due to malignant pheochromocytoma that had invaded into the left kidney eight months prior to presentation. A coronary artery bypass graft operation two years previously and acetylsalicylic acid use were also reported in the medical history. The vital signs were stable, without hypertension and tachycardia. Physical examination revealed abdominal tenderness in the epigastric region, with mild abdominal distention. No abdominal guarding was present, but rebound tenderness occurred. The physical examination also identified hyperkinetic bowel sounds and empty rectal ampulla upon digital examination. Sternotomy and left subcostal incision scars were clearly visible on the patient. A contrast-enhanced abdominal computed tomography (CT) scan was performed. The CT scan revealed the jejunojejunal invagination of a contrast enhancing mass 20×9 mm in size located at the distal jejunum. The jejunum was dilated, resulting in a proximal diameter ≤3.5 cm. The CT findings featured a hypervascular target sign resembling a neuroendocrine tumor (Fig. 1).

**Figure 2:**
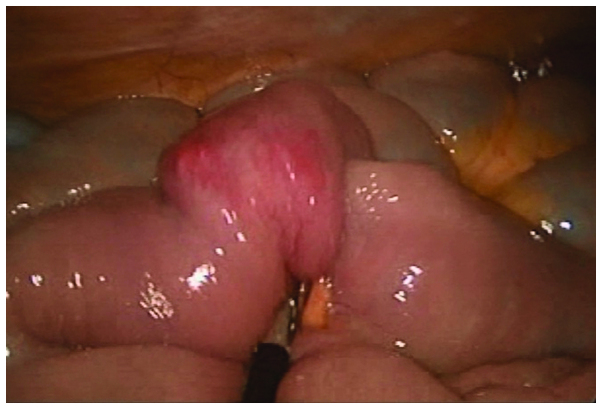
Intra-operative image of the invaginated jejunal segment.

**Figure 3:**
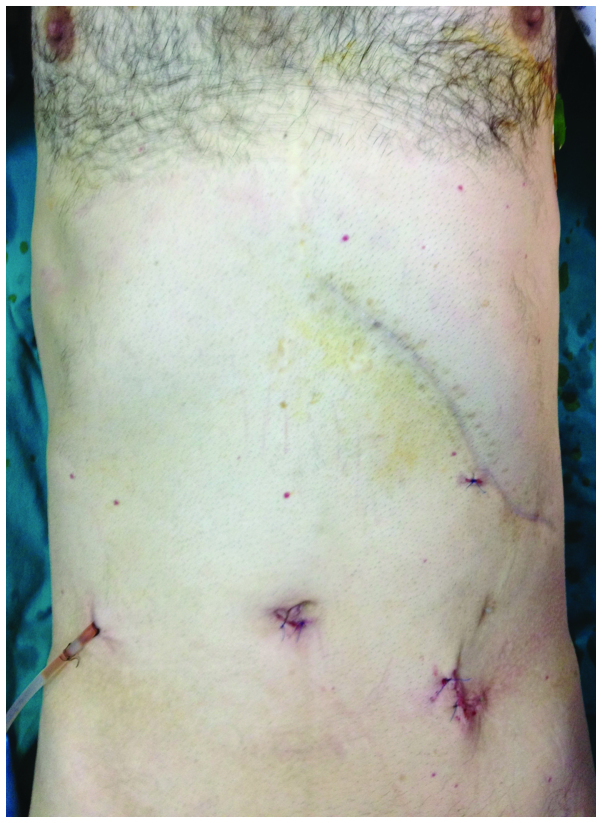
A minimally invasive approach was used in the surgical procedure, which was performed via small incisions. The scar from the previous left subcostal incision can be observed.

**Figure 4:**
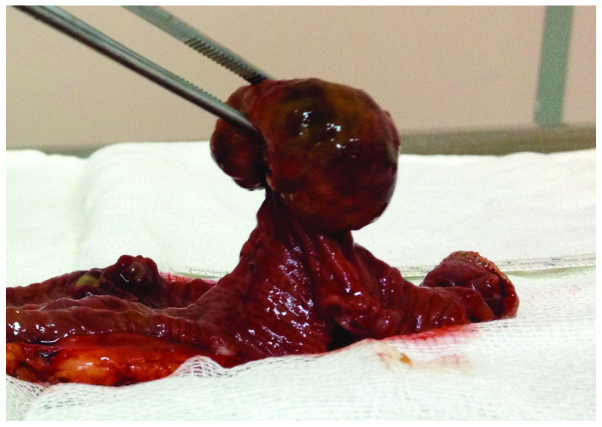
Polypoid mass subsequent to dissection of the specimen.

Informed consent was obtained from the patient prior to undergoing surgery. In the present patient, a laparoscopic approach was preferred. The invaginated jejunal segment was identified at 120 cm distal to the ligament of Treitz (Fig. 2). The segmentary resection was performed laparoscopically to provide a minimally invasive approach and a soft suction drainage tube was inserted into the abdominal cavity (Fig. 3). A polypoid mass 3.5×3.5×2 cm in size was identified subsequent to specimen dissection (Fig. 4).

**Figure 5:**
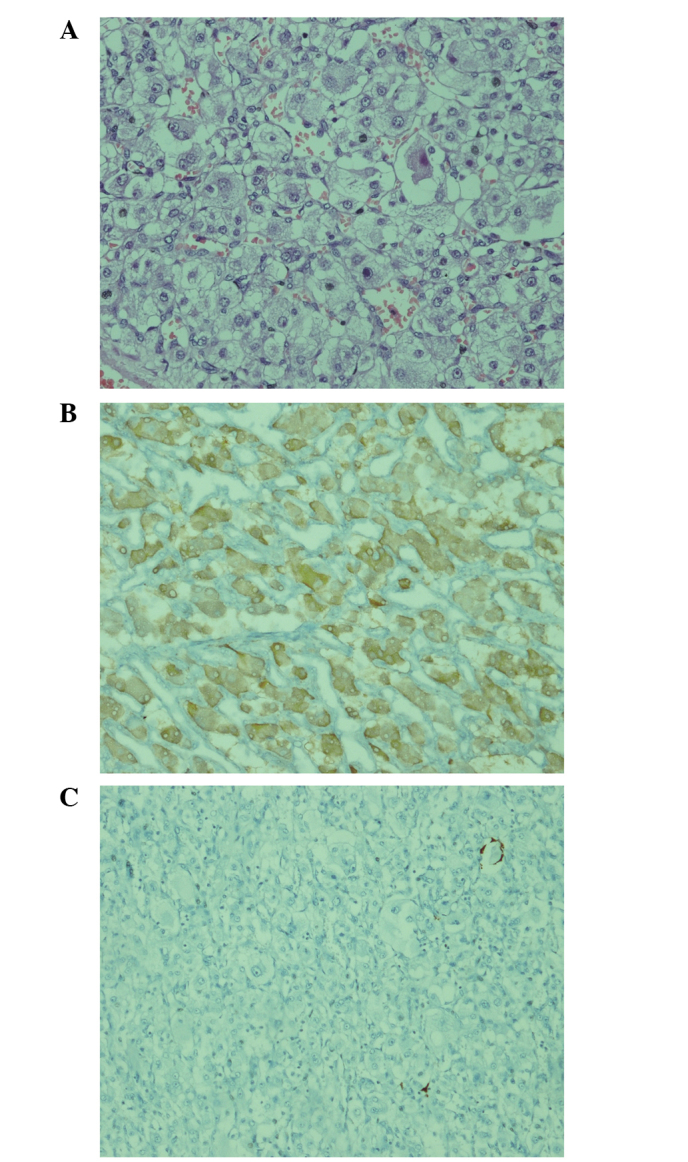
(A) Ganglion-like tumor cells with atypical features similar to large eosinophilic cytoplasm demonstrated positive staining for the expression of (B) neuron-specific enolase, but (C) did not express S-100.

Pathological examination of the mass revealed a malignant mesenchymal neuroendocrine tumor with vascular invasion. Mitosis was identified at a level of three mitoses per 10 high-power fields and the Ki-67 index was 40%. The surgical margins were tumor-free. Immunohistochemistry (IHC) revealed islets of ganglion-like cells that possessed large eosinophilic cytoplasms and were located in the submucosa under the epithelium. These cells were positive for the expression of neuron-specific enolase and did not express S-100 (Fig. 5). The final pathological diagnosis was malignant paraganglioma. No post-operative early complications occurred and the patient was discharged on post-operative day five. It was decided to perform a follow-up without the administration of adjuvant therapy at the oncological council of Bezmialem Vakif University (Istanbul, Turkey). Currently, the patient remains alive without recurrence six months subsequent to the start of the follow-up.

## Discussion

Pheochromocytomas are neuroendocrine tumors that arise from the chromaffin cells of the adrenal medulla. The neuroendocrine tumors that arise from the extra-adrenal paraganglia are termed paraganglioma. Pheochromocytomas are occasionally termed as intra-adrenal paraganglia (1). The incidence of paraganglioma is estimated as 1.5 cases per million individuals each year, and the peak incidence occurs at 30–50 years (2). Paragangliomas can be derived from either parasympathetic or sympathetic ganglia. Paragangliomas derived from parasympathetic ganglia are mostly located in the head and neck, whereas the majority of sympathetic ganglia-derived paragangliomas are located in the abdomen, adjacent to the adrenals (1). However, paragangliomas can be found in any region that the sympathetic or parasympathetic ganglia exist. The present study reports the case of a patient exhibiting paraganglioma that arose from the jejunum of the small intestine, which is an extremely rarely reported location in the literature. Despite the majority of paragangliomas being sporadic, a genetic association has been identified in up to one-half of cases due to the evolution in genetic testing (3). In total, ~25% of paragangliomas are malignant and metastasis may arise≥20 years after the first presentation, which indicates the importance of long-term follow-up (4). In the present case, paraganglioma arose eight months subsequent to the first appearance of a tumor in the left adrenal gland, which invaded into the left kidney. The majority of paragangliomas are chemo- and radioresistant (2). Thus, surgical excision of the tumor is the primary treatment (5,6).

For the surgical excision of paragangliomas, either an open or laparoscopic approach can be performed. In a case of suspected malignancy, the open approach is recommended by certain literature in order to protect the capsule of the tumor and avoid seeding, therefore preventing recurrence (7). However, experienced surgeons are able to perform the laparoscopic approach safely even in emergency settings (8, 9). In the present study, the laparoscopic approach was preferred due to advantages that included a low rate of occurrence of intraperitoneal adhesions, a decrease in the severity of post-operative pain, decreased occurrence of surgical site infections, an earlier recovery and a shorter hospital stay compared with the open approach (10). The five-year survival rate of patients with malignant paragangliomas is ~60% (6). Recurrence is observed within 10 years of the first surgical procedure in 16% of pheochromocytoma or paraganglioma cases (11). Therefore, a long-term follow-up is required.
